# Contemporaneous data on the prevalence of Human Respiratory Syncytial Virus infection in people with acute respiratory tract infections in Africa (2000–2017)

**DOI:** 10.1016/j.dib.2018.08.039

**Published:** 2018-08-22

**Authors:** Jean Joel Bigna, Sebastien Kenmoe, Estelle Amandine Well, Fredy Brice N. Simo, Véronique B. Penlap, Astrid Vabret, Richard Njouom

**Affiliations:** a National Influenza Center, Centre Pasteur of Cameroon, 451 Rue 2005, P.O. Box 1274, Yaoundé, Cameroon; b Faculty of Medicine and Biomedical Sciences, University of Yaoundé 1, P.O. Box 1364, Yaoundé, Cameroon; c Department of Biochemistry, Faculty of Sciences, University of Yaoundé 1, P.O. Box 337, Yaoundé, Cameroon; d Normandie Université, 14032 Caen, France; e Université de Caen, Groupe de Recherche sur l’Adaptation Microbienne (GRAM), F-14000 Caen, France; f Laboratoire de Virologie, Centre Hospitalo-Universitaire de Caen, F-14033 Caen, France

**Keywords:** Human Respiratory Syncytial Virus, Respiratory tract infection, Africa

## Abstract

Availability of accurate data on the burden of the Human Respiratory Syncytial Virus (HRSV) can help to implement better strategies to curb this burden in Africa continent among people with acute respiratory tract infections (ARTI). We summarize here available contemporaneous data published from January 1, 2000 to August 31, 2017 on the prevalence of HSRV infection among people with ARTI in the continent.

## Specifications Table

**Table:** 

**Subject area**	Medicine
**More specific subject area**	Virology, Epidemiology
**Type of data**	Data presented in tables, CSV database, R codes
**How data was acquired**	Systematic search of literature
**Data format**	Raw data
**Experimental factors**	Not applicable
**Experimental features**	Not applicable
**Data source location**	Not applicable
**Data accessibility**	All data are included in this article
**Related research article**	Prevalence of Human Respiratory Syncytial Virus infection in people with acute respiratory tract infections in Africa: a systematic review and meta-analysis [1].

## Value of the data

• This work provides data to understanding the prevalence and distribution of HRSV infection in people with ARTI in Africa.
• The data allow deeper examination of epidemiology of HRSV infection in Africa and therefore could help for better prevention and control for HRSV infection in the continent.
• The data could be used as baseline for comparison in future studies and comparison with data from other regions outside Africa.

## 1 Data

Availability of accurate data on the burden of the Human Respiratory Syncytial Virus (HRSV) can help to implement better strategies to curb this burden in Africa continent among people with acute respiratory tract infections (ARTI). To date, data synthesis on the epidemiology of HRSV infection prevalence in the continent are lacking. We present here a summary of available data on the prevalence based on HRSV infection in people with ARTI in Africa.

## 2 Experimental design, materials and methods

A comprehensive search of PubMed, Excerpta Medica Database, Africa Journals Online, and Global Index Medicus helped to identify all published data from January 1, 2000 and September 18, 2017 on the prevalence of HRSV infection in Africa. The search was limited in the last 18 years to have contemporaneous and relevant data. Table 1 presents the search strategy in PubMed. This search strategy was adapted to fit with other databases. Studies conducted exclusively on African populations living outside Africa, commentaries, editorials, case reports, case series, letters to editor, duplicates, studies lacking prevalence data (number of cases and sample size) on HRSV, and studies lacking full text even after request from authors were excluded. HRSV infection had to be diagnosed with polymerase chain reaction technique on respiratory samples.

**Table 1:** Search strategy.

**Search**	**Search terms**
#1	“HRSV” OR “RSV” OR “human respiratory syncytial virus” OR “respiratory syncytial virus”
#2	“respiratory tract infections” OR “respiratory tract infection” OR “respiratory infection” OR “respiratory infections” OR “lower respiratory tract infections” OR “LRTI” OR “acute lower respiratory infections” OR “ALRI” OR “pneumonia” OR “community acquired pneumonia” OR “bronchiolitis” OR “severe acute respiratory infections” OR “severe acute respiratory illness” OR “experimental lung inflammation” OR “pneumonitis” OR “pulmonary inflammation” OR “bronchopneumonia” OR “pleuropneumonia”
#3	Africa* OR Algeria OR Angola OR Benin OR Botswana OR "Burkina Faso" OR Burundi OR Cameroon OR "Canary Islands" OR "Cape Verde" OR "Central African Republic" OR Chad OR Comoros OR Congo OR "Democratic Republic of Congo" OR Djibouti OR Egypt OR "Equatorial Guinea" OR Eritrea OR Ethiopia OR Gabon OR Gambia OR Ghana OR Guinea OR "Guinea Bissau" OR "Ivory Coast" OR "Cote d׳Ivoire" OR Jamahiriya OR Kenya OR Lesotho OR Liberia OR Libya OR Madagascar OR Malawi OR Mali OR Mauritania OR Mauritius OR Mayotte OR Morocco OR Mozambique OR Namibia OR Niger OR Nigeria OR Principe OR Reunion OR Rwanda OR "Sao Tome" OR Senegal OR Seychelles OR "Sierra Leone" OR Somalia OR "South Africa" OR “South Sudan” OR "St Helena" OR Sudan OR Swaziland OR Tanzania OR Togo OR Tunisia OR Uganda OR "Western Sahara" OR Zaire OR Zambia OR Zimbabwe OR "Central Africa" OR "Central African" OR "West Africa" OR "West African" OR "Western Africa" OR "Western African" OR "East Africa" OR "East African" OR "Eastern Africa" OR "Eastern African" OR "North Africa" OR "North African" OR "Northern Africa" OR "Northern African" OR "South African" OR "Southern Africa" OR "Southern African" OR "sub Saharan Africa" OR "sub Saharan African" OR "sub Saharan Africa" OR "sub Saharan African”
#4	#1 AND #2 AND #3
#5	Limits 2000/01/01–2017/08/31

Titles and abstracts of all records were reviewed by two investigators and full texts of eligible records were assessed. Reference lists of eligible papers and relevant review articles were scanned to identify other eligible papers. Disagreements were solved through a discussion or by an arbitration of a third investigator. In total, 66 full texts including 67 studies were retained (one paper included two studies) [2], [3], [4], [5], [6], [7], [8], [9], [10], [11], [12], [13], [14], [15], [16], [17], [18], [19], [20], [21], [22], [23], [24], [25], [26], [27], [28], [29], [30], [31], [32], [33], [34], [35], [36], [37], [38], [39], [40], [41], [42], [43], [44], [45], [46], [47], [48], [49], [50], [51], [52], [53], [54], [55], [56], [57], [58], [59], [60], [61], [62], [63], [64], [65], [66], [67]. Table 2 presents the risk of bias in each included study using an 8-item rating scale [68]. Disagreements were solved through discussion and consensus.

**Table 2:** Risk of bias in individual included studies.

**Study**	**Item 1**	**Item 2**	**Item 3**	**Item 4**	**Item 5**	**Item 6**	**Item 7**	**Item 8**	**Score**	**Bias**
**Agoti**[2]	1	1	0	1	0	1	0	1	**5**	Moderate risk
**Ahmed**[3]	1	1	1	1	1	1	1	1	**8**	Low risk
**Akinloye**[4]	0	1	1	1	1	1	0	1	**6**	Low risk
**Annamalay**[5]	0	1	1	1	1	0	0	1	**5**	Moderate risk
**Berkley**[6]	1	1	1	1	1	1	1	1	**8**	Low risk
**Bigogo**[58]	1	1	1	1	1	1	1	1	**8**	Low risk
**Bimouhen**[7]	1	1	1	1	1	1	1	1	**8**	Low risk
**Breiman**[8]	1	1	1	1	1	1	1	1	**8**	Low risk
**Brottet**[59]	1	1	1	0	1	1	0	0	**5**	Moderate risk
**Ciervo**[9]	1	1	1	1	1	1	0	1	**7**	Low risk
**Cohen**[10]	0	1	1	1	1	1	1	1	**7**	Low risk
**Cohen**[46]	0	1	1	1	1	1	0	1	**7**	Low risk
**Dia**[47]	1	1	1	1	0	1	1	1	**6**	Low risk
**Dia**[60]**(2)**	1	1	1	1	1	1	1	1	**8**	Low risk
**El Kholy**[12]	1	1	1	1	1	1	1	1	**8**	Low risk
**ElBasha**[11]	0	1	0	0	1	1	1	1	**5**	Moderate risk
**Embarek Mohamed**[61]	1	1	1	1	1	1	1	0	**7**	Low risk
**Emukule**[13]	0	1	1	0	1	1	1	1	**6**	Low risk
**Enan**[14]	0	1	1	1	1	1	1	1	**7**	Low risk
**Fall**[48]	1	1	1	1	1	1	1	1	**7**	Low risk
**Feikin**[16]	0	1	1	1	1	1	1	1	**7**	Low risk
**Feikin**[16]**(2)**	1	1	1	1	1	1	1	0	**7**	Low risk
**Feikin**[15]**(1)**	0	1	1	1	1	1	1	1	**7**	Low risk
**Feikin**[62]**(2)**	0	1	1	1	1	1	1	1	**7**	Low risk
**Fuller**[17]	0	1	1	1	1	1	1	1	**7**	Low risk
**Ghani**[18]	0	0	1	1	0	1	0	1	**4**	Moderate risk
**Hammitt**[19]	0	1	1	1	1	1	1	1	**7**	Low risk
**Hoffman**[20]	0	1	1	1	1	1	1	1	**7**	Low risk
**Horton**[21]	1	1	1	1	1	1	1	1	**8**	Low risk
**Jroundi**[22]	1	1	1	1	1	1	1	1	**8**	Low risk
**Jroundi**[63]	0	1	1	1	1	1	0	1	**6**	Low risk
**Kadjo**[49]	0	1	1	1	1	1	0	1	**8**	Low risk
**Kelly**[23]	0	1	1	1	1	1	0	1	**6**	Low risk
**Kenmoe**[24]	1	1	1	1	1	1	1	1	**8**	Low risk
**Kim**[25]	0	1	1	1	0	1	1	1	**6**	Low risk
**Kwofie**[26]	1	1	1	1	1	1	1	1	**8**	Low risk
**Lagare**[27]	1	1	1	1	1	1	1	1	**8**	Low risk
**Lekana-Douki**[50]	1	1	1	1	1	1	1	1	**7**	Low risk
**Lonngren**[28]	0	1	1	1	1	1	1	1	**7**	Low risk
**Mazur**[29]	1	1	1	1	1	1	1	1	**8**	Low risk
**Meligy**[30]	0	1	1	1	1	1	0	1	**6**	Low risk
**Mohamed**[64]	0	1	1	1	1	1	0	1	**6**	Low risk
**Moyes**[51]	1	1	1	1	1	1	1	1	**4**	Low risk
**Moyes**[52]	1	1	1	1	1	1	0	1	**8**	Low risk
**Nakouné**[53]	1	1	1	1	1	1	1	1	**7**	Low risk
**Ndegwa**[54]	1	0	1	1	1	1	1	1	**8**	Low risk
**Niang**[31]	0	0	1	1	1	1	0	1	**5**	Moderate risk
**Niang**[55]	1	1	1	1	1	1	0	1	**8**	Low risk
**Njouom**[57]	1	1	1	1	0	1	1	1	**6**	Low risk
**Nyawanda**[65]	1	1	1	1	1	1	1	1	**8**	Low risk
**Obodai**[32]	1	1	1	1	1	1	0	1	**7**	Low risk
**O׳Callaghan-Gordo**[33]	1	1	1	1	1	1	1	1	**8**	Low risk
**Othman**[34]	1	1	1	1	1	1	1	1	**8**	Low risk
**Otieno**[35]	1	1	1	1	1	1	1	1	**8**	Low risk
**Ouédraogo Yugbaré**[66]	1	1	1	1	1	1	1	1	**8**	Low risk
**Ouedraogo**[36]	0	1	1	1	1	1	0	1	**6**	Low risk
**Peterson**[37]	0	1	1	1	1	1	0	1	**6**	Low risk
**Pretorius**[38]	1	1	1	1	0	1	1	1	**7**	Low risk
**Pretorius**[39]	1	1	1	1	0	1	1	1	**7**	Low risk
**Pretorius**[40]	1	1	1	1	0	1	1	1	**7**	Low risk
**Razanajatovo**[57]	0	1	1	0	0	1	0	1	**0**	Moderate risk
**Rowlinson**[41]	0	1	1	1	1	1	1	1	**7**	Low risk
**Rowlinson**[42]	0	1	1	1	1	1	0	1	**6**	Low risk
**Shafik**[43]	1	1	1	1	1	1	1	1	**8**	Low risk
**Simusika**[67]	1	1	1	1	1	1	0	1	**7**	Low risk
**Venter**[44]	1	1	1	1	1	1	0	1	**7**	Low risk
**Zar**[45]	1	1	1	1	1	1	0	1	**7**	Low risk

Extracted data from original studies included [2], [3], [4], [5], [6], [7], [8], [9], [10], [11], [12], [13], [14], [15], [16], [17], [18], [19], [20], [21], [22], [23], [24], [25], [26], [27], [28], [29], [30], [31], [32], [33], [34], [35], [36], [37], [38], [39], [40], [41], [42], [43], [44], [45], [46], [47], [48], [49], [50], [51], [52], [53], [54], [55], [56], [57], [58], [59], [60], [61], [62], [63], [64], [65], [66], [67]: first author name, year of publication, design, setting, sampling method, respiratory samples collection period, timing of data analysis, number of viruses screened, site of recruitment location (country, city, latitude, longitude, and altitude), clinical presentation, number of patients screened, number of patients infected with HRSV, diagnostic techniques used, and proportion of male participants. We assigned a United Nations Statistics Division (UNSD) African region (Central, Eastern, Northern, Southern, and Western) to each study regarding the country of recruitment [69]. We considered two groups of clinical presentation: severe respiratory tract infection (SRTI) and benign respiratory tract infection (BRTI). Using Google Global Positioning System, we assigned altitude, latitude and longitude according to the cities and country of recruitment [70]. In the case of multi-cities, we considered the median. All these data are presented in Supplementary Table 3. These data are related to a systematic review and meta-analysis published in Influenza and Other Respiratory Viruses [1]. The CSV database used for meta-analysis is online alongside with R codes used.

Risk of bias in included studies using an 8-item rating scale [68]. These items included: (item 1) participation response rate more than 75% agree to participate or analysis to show whether respondents and non-respondents were similar for the sociodemographic characteristics; (Item 2) acute respiratory tract infection clearly defined; (item 3) method of inclusion identical for all subjects; (item 4) description of diagnostic technique; (item 5) same type of sample collected for all patients (nasopharyngeal aspirate, nasal or throat swab); (item 6) standardized method for sample collection (quantity of aspirate or of liquid used for the nasal wash with any virological medium transport for swabs); (item 7) analysis performed according to relevant subgroups (by age classes, by center, or by symptomatology, for example); (item 8) and presentation of data sources (counts are presented, not only percentages).

Each item was assigned a score of 1 (Yes) or 0 (No), and each score was summed across items to generate an overall study quality score. The total score was ranged from 0 to 8 with the overall score categorized as follows: 6–8: “low risk of bias”, 3–5: “moderate risk”, and 0–2: “high risk”.

## References

[1] S. Kenmoe J.J. Bigna E.A. Well F.B.N. Simo V.B. Penlap A. Vabret Prevalence of Human Respiratory Syncytial Virus infection in people with acute respiratory tract infections in Africa: a systematic review and meta-analysis *Influenza Other Respir. Viruses* 2018, 10.1111/irv.12584 (In Press) PMC6185896 29908103

[2] Agoti C.N., Otieno J.R., Gitahi C.W., Cane P.A., Nokes D.J. (2014). Rapid spread and diversification of respiratory syncytial virus genotype ON1, Kenya. Emerg. Infect. Dis..

[3] Ahmed J.A., Katz M.A., Auko E., Njenga M.K., Weinberg M., Kapella B.K. (2012). Epidemiology of respiratory viral infections in two long-term refugee camps in Kenya, 2007–2010. BMC Infect. Dis..

[4] Akinloye O.M., Rönkkö E., Savolainen-Kopra C., Ziegler T., Iwalokun B.A., Deji-Agboola M.A. (2011). Specific viruses detected in Nigerian children in association with acute respiratory disease. J. Trop. Med..

[5] Annamalay A., Lanaspa M., Bizzintino J., Khoo S., Le Souef P., Bassat Q. (2014). Viral aetiology of acute lower respiratory infections in children in rural mozambique. Respirology.

[6] Berkley J.A., Munywoki P., Ngama M., Kazungu S., Abwao J., Bett A. (2010). Viral etiology of severe pneumonia among Kenyan infants and children. Jama.

[7] Bimouhen A., El Falaki F., Ihazmad H., Regragui Z., Benkerroum S., Barakat A. (2016). Circulation of Respiratory Syncytial Virus in Morocco during 2014–2016: findings from a sentinel-based virological surveillance system for influenza. East. Mediterr. Health J. = Rev. sante Mediterr. Orient. = al-Majallah al-sihhiyah li-sharq al-mutawassit.

[8] Breiman R.F., Cosmas L., Njenga M., Williamson J., Mott J.A., Katz M.A. (2015). Severe acute respiratory infection in children in a densely populated urban slum in Kenya, 2007–2011. BMC Infect. Dis..

[9] Ciervo A., Mancini F., Puzelli S., Interisano M., Vescio M.F., Farchi F. (2010). Detection and correlates of Chlamydophila pneumoniae among children with acute respiratory infections. J. Pediatr. Infect. Dis..

[10] Cohen C., Walaza S., Moyes J., Groome M., Tempia S., Pretorius M. (2015). Epidemiology of viral-associated acute lower respiratory tract infection among children < 5 years of age in a high HIV prevalence setting, South Africa, 2009–2012. Pediatr. Infect. Dis. J..

[11] ElBasha N., El Rifai N., Draz I., El Kholy A. (2013). Contribution of viruses to severe pneumonia in children. Egypt. Pediatr. Assoc. Gaz..

[12] El Kholy A.A., Mostafa N.A., Ali A.A., El-Sherbini S.A., Ismail R.I., Magdy R.I. (2014). Risk factors of prolonged hospital stay in children with viral severe acute respiratory infections. J. Infect. Dev. Ctries..

[13] Emukule G.O., Khagayi S., McMorrow M.L., Ochola R., Otieno N., Widdowson M.A. (2014). The burden of influenza and RSV among inpatients and outpatients in rural western Kenya, 2009–2012. PLoS One.

[14] Enan K.A., Nabeshima T., Kubo T., Buerano C.C., El Hussein A.R., Elkhidir I.M. (2013). Survey of causative agents for acute respiratory infections among patients in Khartoum-State, Sudan, 2010–2011. Virol. J..

[15] Feikin D.R., Njenga M.K., Bigogo G., Aura B., Aol G., Audi A. (2013). Viral and bacterial causes of severe acute respiratory illness among children aged less than 5 years in a high malaria prevalence area of western Kenya, 2007–2010. Pediatr. Infect. Dis. J..

[16] Feikin D.R., Njenga M.K., Bigogo G., Aura B., Aol G., Audi A. (2012). Etiology and Incidence of viral and bacterial acute respiratory illness among older children and adults in rural western Kenya, 2007–2010. PLoS One.

[17] Fuller J.A., Njenga M.K., Bigogo G., Aura B., Ope M.O., Nderitu L. (2013). Association of the CT values of real-time PCR of viral upper respiratory tract infection with clinical severity, Kenya. J. Med. Virol..

[18] Ghani A.S., Morrow B.M., Hardie D.R., Argent A.C. (2012). An investigation into the prevalence and outcome of patients admitted to a pediatric intensive care unit with viral respiratory tract infections in Cape Town, South Africa. Pediatr. Crit..

[19] Hammitt L.L., Kazungu S., Morpeth S.C., Gibson D.G., Mvera B., Brent A.J. (2012). A preliminary study of pneumonia etiology among hospitalized children in Kenya. Clin. Infect. Dis.: Off. Publ. Infect. Dis. Soc. Am..

[20] Hoffmann J., Rabezanahary H., Randriamarotia M., Ratsimbasoa A., Najjar J., Vernet G. (2012). Viral and atypical bacterial etiology of acute respiratory infections in children under 5 years old living in a rural tropical area of Madagascar. PLoS One.

[21] Horton K.C., Dueger E.L., Kandeel A., Abdallat M., El-Kholy A., Al-Awaidy S. (2017). Viral etiology, seasonality and severity of hospitalized patients with severe acute respiratory infections in the Eastern Mediterranean Region, 2007–2014. PLoS One.

[22] Jroundi I., Mahraoui C., Benmessaoud R., Moraleda C., Tligui H., Seffar M. (2014). The epidemiology and aetiology of infections in children admitted with clinical severe pneumonia to a university hospital in Rabat, Morocco. J. Trop. Pediatr..

[23] Kelly M.S., Smieja M., Luinstra K., Wirth K.E., Goldfarb D.M., Steenhoff A.P. (2015). Association of respiratory viruses with outcomes of severe childhood pneumonia in Botswana. PLoS One.

[24] Kenmoe S., Tchendjou P., Vernet M.A., Moyo-Tetang S., Mossus T., Njankouo-Ripa M. (2016). Viral etiology of severe acute respiratory infections in hospitalized children in Cameroon, 2011–2013. Influenza Other Respir. Viruses.

[25] Kim C., Ahmed J.A., Eidex R.B., Nyoka R., Waiboci L.W., Erdman D. (2011). Comparison of nasopharyngeal and oropharyngeal swabs for the diagnosis of eight respiratory viruses by real-time reverse transcription-PCR assays. PLoS One.

[26] Kwofie T.B., Anane Y.A., Nkrumah B., Annan A., Nguah S.B., Owusu M. (2012). Respiratory viruses in children hospitalized for acute lower respiratory tract infection in Ghana. Virol. J..

[27] Lagare A., Mainassara H.B., Issaka B., Sidiki A., Tempia S. (2015). Viral and bacterial etiology of severe acute respiratory illness among children < 5 years of age without influenza in Niger. BMC Infect. Dis..

[28] Lonngren C., Morrow B.M., Haynes S., Yusri T., Vyas H., Argent A.C. (2014). North-South divide: distribution and outcome of respiratory viral infections in paediatric intensive care units in Cape Town (South Africa) and Nottingham (United Kingdom). J. Paediatr. Child Health.

[29] Mazur N.I., Bont L., Cohen A.L., Cohen C., von Gottberg A., Groome M.J. (2017). Severity of respiratory Syncytial Virus Lower respiratory tract infection with viral coinfection in HIV-uninfected children. Clin. Infect. Dis.: Off. Publ. Infect. Dis. Soc. Am..

[30] Meligy B., Sayed A., Ismail D.K., Kamal D., Abdel-Latif W., Erfan D.M. (2016). Detection of viral acute lower respiratory tract infection in hospitalized infants using real-time PCR. Egypt. Pediatr. Assoc. Gaz..

[31] Niang M.N., Diop O.M., Sarr F.D., Goudiaby D., Malou-Sompy H., Ndiaye K. (2010). Viral etiology of respiratory infections in children under 5 years old living in tropical rural areas of Senegal: the EVIRA project. J. Med. Virol..

[32] Obodai E., Asmah R., Boamah I., Goka B., Odoom J.K., Adiku T. (2014). Respiratory syncytial virus genotypes circulating in urban Ghana: february–november 2006. Pan Afr. Med. J..

[33] O׳Callaghan-Gordo C., Bassat Q., Morais L., Diez-Padrisa N., Machevo S., Nhampossa T. (2011). Etiology and epidemiology of viral pneumonia among hospitalized children in rural Mozambique: a malaria endemic area with high prevalence of human immunodeficiency virus. Pediatr. Infect. Dis. J..

[34] Othman H.T., Abu Elhamed W.A., Hassan D.M., Soliman M.S., Abdel Baset R.W. (2016). Respiratory syncytial virus and human metapneumovirus in severe lower respiratory tract infections in children under two. J. Infect. Dev. Ctries..

[35] Otieno J.R., Kamau E.M., Agoti C.N., Lewa C., Otieno G., Bett A. (2017). Spread and evolution of respiratory syncytial virus A genotype ON1, Coastal Kenya, 2010–2015. Emerg. Infect. Dis..

[36] Ouedraogo S., Traore B., Nene Bi Z.A., Yonli F.T., Kima D., Bonane P. (2014). Viral etiology of respiratory tract infections in children at the pediatric hospital in Ouagadougou (Burkina Faso). PLoS One.

[37] Peterson I., Bar-Zeev N., Kennedy N., Ho A., Newberry L., Joaquin M.A.S. (2016). Respiratory virus-associated severe acute respiratory illness and viral clustering in malawian children in a setting with a high prevalence of HIV infection, malaria, and malnutrition. J. Infect. Dis..

[38] Pretorius M.A., Madhi S.A., Cohen C., Naidoo D., Groome M., Moyes J. (2012). Respiratory viral coinfections identified by a 10-plex real-time reverse-transcription polymerase chain reaction assay in patients hospitalized with severe acute respiratory illness – South Africa, 2009–2010. J. Infect. Dis..

[39] Pretorius M.A., van Niekerk S., Tempia S., Moyes J., Cohen C., Madhi S.A. (2013). Replacement and positive evolution of subtype A and B respiratory syncytial virus G-protein genotypes from 1997–2012 in South Africa. J. Infect. Dis..

[40] Pretorius M.A., Tempia S., Walaza S., Cohen A.L., Moyes J., Variava E. (2016). The role of influenza, RSV and other common respiratory viruses in severe acute respiratory infections and influenza-like illness in a population with a high HIV sero-prevalence, South Africa 2012–2015. J. Clin. Virol.: Off. Publ. Pan Am. Soc. Clin. Virol..

[41] Rowlinson E., Dueger E., Taylor T., Mansour A., Van Beneden C., Abukela M. (2013). Incidence and clinical features of respiratory syncytial virus infections in a population-based surveillance site in the Nile Delta Region. J. Infect. Dis..

[42] Rowlinson E., Dueger E., Mansour A., Azzazy N., Mansour H., Peters L. (2017). Incidence and etiology of hospitalized acute respiratory infections in the Egyptian Delta. Influenza Other Respir. Viruses.

[43] Shafik C.F., Mohareb E.W., Yassin A.S., Amin M.A., El Kholy A., El-Karaksy H. (2012). Viral etiologies of lower respiratory tract infections among Egyptian children under five years of age. BMC Infect. Dis..

[44] Venter M., Lassauniere R., Kresfelder T.L., Westerberg Y., Visser A. (2011). Contribution of common and recently described respiratory viruses to annual hospitalizations in children in South Africa. J. Med. Virol..

[45] Zar H.J., Barnett W., Stadler A., Gardner-Lubbe S., Myer L., Nicol M.P. (2016). Aetiology of childhood pneumonia in a well vaccinated South African birth cohort: a nested case-control study of the Drakenstein Child Health Study. Lancet Respir. Med..

[46] Cohen C., Moyes J., Tempia S., Groome M., Walaza S., Pretorius M. (2016). Epidemiology of acute lower respiratory tract infection in HIV exposed uninfected infants. Pediatrics.

[47] Dia N., Richard V., Kiori D., Cisse el H.A., Sarr F.D., Faye A. (2014). Respiratory viruses associated with patients older than 50 years presenting with ILI in Senegal, 2009–2011. BMC Infect. Dis..

[48] Fall A., Dia N., Cisse el H.A., Kiori D.E., Sarr F.D., Sy S. (2016). Epidemiology and molecular characterization of human respiratory syncytial virus in Senegal after four consecutive years of surveillance, 2012–2015. PLoS One.

[49] Kadjo H.A., Ekaza E., Coulibaly D., Kouassi D.P., Nzussouo N.T., Kouakou B. (2013). Sentinel surveillance for influenza and other respiratory viruses in Cote d׳Ivoire, 2003–2010. Influenza Other Respir. Viruses.

[50] Lekana-Douki S.E., Nkoghe D., Drosten C., Ngoungou E.B., Drexler J.F., Leroy E.M. (2014). Viral etiology and seasonality of influenza-like illness in Gabon, March 2010–June 2011. BMC Infect. Dis..

[51] Moyes J., Cohen C., Pretorius M., Groome M., von Gottberg A., Wolter N. (2013). Epidemiology of respiratory syncytial virus-associated acute lower respiratory tract infection hospitalizations among HIV-infected and HIV-uninfected South African children, 2010–2011. J. Infect. Dis..

[52] Moyes J., Walaza S., Pretorius M., Groome M., von Gottberg A., Wolter N. (2017). Respiratory syncytial virus in adults with severe acute respiratory illness in a high HIV prevalence setting. J. Infect..

[53] Nakoune E., Tricou V., Manirakiza A., Komoyo F., Selekon B., Gody J.C. (2013). First introduction of pandemic influenza A/H1N1 and detection of respiratory viruses in pediatric patients in Central African Republic. Virol. J..

[54] Ndegwa L.K., Katz M.A., McCormick K., Nganga Z., Mungai A., Emukule G. (2014). Surveillance for respiratory health care-associated infections among inpatients in 3 Kenyan hospitals, 2010–2012. Am. J. Infect. Control.

[55] Niang M.N., Diop N.S., Fall A., Kiori D.E., Sarr F.D., Sy S. (2017). Respiratory viruses in patients with influenza-like illness in Senegal: focus on human respiratory adenoviruses. PLoS One.

[56] Njouom R., Yekwa E.L., Cappy P., Vabret A., Boisier P., Rousset D. (2012). Viral etiology of influenza-like illnesses in Cameroon, January–December 2009. J. Infect. Dis..

[57] Razanajatovo N.H., Richard V., Hoffmann J., Reynes J.M., Razafitrimo G.M., Randremanana R.V. (2011). Viral etiology of influenza-like illnesses in Antananarivo, Madagascar, July 2008–June 2009. PLoS One.

[58] Bigogo G.M., Breiman R.F., Feikin D.R., Audi A.O., Aura B., Cosmas L. (2013). Epidemiology of respiratory syncytial virus infection in rural and urban Kenya. J. Infect. Dis..

[59] Brottet E., Jaffar-Bandjee M.C., Li-Pat-Yuen G., Filleul L. (2016). Etiology of influenza-like illnesses from sentinel network practitioners in Reunion Island, 2011–2012. PLoS One.

[60] Dia N., Diene Sarr F., Thiam D., Faye Sarr T., Espie E., OmarBa I. (2014). Influenza-like illnesses in Senegal: not only focus on influenza viruses. PLoS One.

[61] Embarek Mohamed M.S., Reiche J., Jacobsen S., Thabit A.G., Badary M.S., Brune W. (2014). Molecular analysis of human metapneumovirus detected in patients with lower respiratory tract infection in upper Egypt. Int. J. Microbiol..

[62] Feikin D.R., Njenga M.K., Bigogo G., Aura B., Gikunju S., Balish A. (2013). Additional diagnostic yield of adding serology to PCR in diagnosing viral acute respiratory infections in Kenyan patients 5 years of age and older. Clin. vaccine Immunol.: CVI.

[63] Jroundi I., Mahraoui C., Benmessaoud R., Moraleda C., Tligui H., Seffar M. (2016). A comparison of human metapneumovirus and respiratory syncytial virus WHO-defined severe pneumonia in Moroccan children. Epidemiol. Infect..

[64] Mohamed G.A., Ahmed J.A., Marano N., Mohamed A., Moturi E., Burton W. (2015). Etiology and incidence of viral acute respiratory infections among refugees aged 5 years and older in Hagadera Camp, Dadaab, Kenya. Am. J. Trop. Med. Hyg..

[65] Nyawanda B.O., Mott J.A., Njuguna H.N., Mayieka L., Khagayi S., Onkoba R. (2016). Evaluation of case definitions to detect respiratory syncytial virus infection in hospitalized children below 5 years in Rural Western Kenya, 2009–2013. BMC Infect. Dis..

[66] Ouedraogo Yugbare S.O., Ouedraogo R., Nenebi A., Traore B., Congo L., Yonli F. (2016). Respiratory syncytial virus (RSV) infections in the pediatric teaching hospital Charles de Gaulle of Ouagadougou, Burkina Faso. Bull. Soc. Pathol. Exot. (1990).

[67] Simusika P., Bateman A.C., Theo A., Kwenda G., Mfula C., Chentulo E. (2015). Identification of viral and bacterial pathogens from hospitalized children with severe acute respiratory illness in Lusaka, Zambia, 2011–2012: a cross-sectional study. BMC Infect. Dis..

[68] Lefebvre A., Manoha C., Bour J.B., Abbas R., Fournel I., Tiv M. (2016). Human metapneumovirus in patients hospitalized with acute respiratory infections: a meta-analysis. J. Clin. Virol.: Off. Publ. Pan Am. Soc. Clin. Virol..

[69] Statistics Division United Nations, Geographic Regions: Statistics Division United Nations; [cited 2017 Sep 1]. Available from: 〈https://unstats.un.org/unsd/methodology/m49/〉.

[70] Google, Coordonnées GPS et Google Map [cited 2017 Sep 1]. Available from: 〈https://www.coordonnees-gps.fr/〉.

