# Supplementary material for: Contemporaneous data on the prevalence of Human Respiratory Syncytial Virus infection in people with acute respiratory tract infections in Africa (2000–2017)
Source: Data Brief. 2018 Aug 22;20:940–7. doi: 10.1016/j.dib.2018.08.039 (PMC6138983; doi:10.1016/j.dib.2018.08.039)
Supplement: Supplementary file 4 — Supplementary material [file mmc4.docx]

**Table 3. Data of included studies**

| Study | Sample | Cases | Prevalence | Design | Setting | Sampling | Period | Timing | Population | %Children | Country | Latitude | Longitude | Altitude | Diagnostic | Samples | %Male |
| --- | --- | --- | --- | --- | --- | --- | --- | --- | --- | --- | --- | --- | --- | --- | --- | --- | --- |
| Agoti, 2013 | 834 | 240 | 28.8% | Cross sectional | Urban and Rural | Consecutive | Jan/2012-Dec/2012 | Prospective | Children | 100 | Kenya | 3.510651 | 39.90932 | 5 | Real time RT-PCR | Nasopharyngeal |  |
| Ahmed, 2012 | 6264 | 781 | 12.5% | Cross sectional | Unclear/Not described | Systematic | Sep/2007-Aug/2010 | Prospective | Children and Adults | 82 | Kenya | 0.09258 | 40.319072 | 120 | Real time RT-PCR | Nasopharyngeal and Oropharyngeal | 54.2 |
| Akinloye, 2011 | 246 | 1 | 0.4% | Cross sectional | Unclear/Not described | Consecutive | Feb/2009-May/2009 | Prospective | Children and Adults | 86 | Nigeria | 7.377536 | 3.94704 | 225 | Real time RT-PCR | Throat and Nasal |  |
| Annamalay, 2016 | 105 | 28 | 26.7% | Cross sectional | Unclear/Not described | Consecutive | Jul/2011-Nov/2012 | Prospective | Children | 100 | Mozambique | -25.392301 | 32.797165 | 31 | Real time RT-PCR | Nasopharyngeal | 67.6 |
| Berkley, 2010 | 759 | 260 | 34.3% | Case control | Rural | Consecutive | Jan/2007-Dec/2007 | Prospective | Children | 100 | Kenya | 3.510651 | 39.90932 | 5 | Real time RT-PCR | Nasal | 59 |
| Bigogo, 2013 | 4012 | 501 | 12.5% | Cross sectional | Urban and Rural | Systematic | Mar/2007-Feb/2011 | Prospective | Children and Adults | 92 | Kenya | -0.691883 | 35.7949515 | 1175 | Real time RT-PCR | Nasopharyngeal and Oropharyngeal | 48.7 |
| Bimouhen, 2016 | 1450 | 267 | 18.4% | Cross sectional | Urban and Rural | Systematic | Sept/2014-Apr/2016 | Prospective | Children and Adults | 45 | Morocco | 33.873016 | -6.849813 | 78 | Real time RT-PCR | Nasopharyngeal and Oropharyngeal | 47.8 |
| Breiman, 2015 | 815 | 169 | 20.7% | Cross sectional | Urban and Rural | Consecutive | Mar/2007–Feb/2011 | Prospective | Children | 100 | Kenya | 48.838235 | 2.337097 | 58 | Real time RT-PCR | Nasopharyngeal and Oropharyngeal | 50.4 |
| Brottet, 2016 | 222 | 6 | 2.7% | Cross sectional | Urban and Rural | Random | 2011-2012 | Retrospective | Children and Adults | na | Réunion | -21.115141 | 55.536384 | 1666 | Real time RT-PCR | Nasal |  |
| Ciervo, 2010 | 113 | 8 | 7.1% | Cross sectional | Urban and Rural | Consecutive | Feb/2007-Apr/2007 | Prospective | Children | 100 | Egypt | 31.200092 | 29.918739 | 9 | Real time RT-PCR | Throat | 63 |
| Cohen, 2015 | 8723 | 1119 | 12.8% | Cross sectional | Urban and Rural | Systematic | Feb/2009-Dec/2012 | Prospective | Children | 100 | South Africa | -26.244583 | 30.379412 | 857 | Real time RT-PCR | Nasopharyngeal |  |
| Cohen, 2016 | 1410 | 469 | 33.3% | Cross sectional | Urban and Rural | Consecutive | Jan/2010-Dec/2013 | Prospective | Children | 100 | South Africa | -26.244583 | 30.379412 | 857 | Real time RT-PCR | Nasopharyngeal |  |
| Dia, 2014 | 232 | 7 | 3.0% | Cross sectional | Unclear/Not described | Consecutive | Jan/2009-Dec/2011 | Prospective | Adults | 0 | Senegal | 14.497401 | -14.452362 | 49 | Real time RT-PCR | Nasopharyngeal and Oropharyngeal | 44.2 |
| Dia, 2014 (2) | 1427 | 158 | 11.1% | Cross sectional | Urban and Rural | Consecutive | Mar/2012-Jul/2013 | Prospective | Children and Adults | 56.3 | Sénégal | 14.497401 | -14.452362 | 49 | Real time RT-PCR | Nasopharyngeal and Oropharyngeal |  |
| El Kholy, 2014 | 1046 | 240 | 22.9% | Cross sectional | Urban | Consecutive | Feb/2010-May/2011 | Prospective | Children | 100 | Egypt | 30.04442 | 31.235712 | 26 | Real time RT-PCR | Nasopharyngeal and Oropharyngeal |  |
| ElBasha, 2013 | 733 | 197 | 26.9% | Cross sectional | Urban | Consecutive | Mar/2010-Apr/2011 | Prospective | Children | 100 | Egypt | 30.04442 | 31.235712 | 26 | Real time RT-PCR | Nasopharyngeal and Oropharyngeal |  |
| Embarek Mohamed, 2014 | 520 | 16 | 3.1% | Cross sectional | Urban and Rural | Consecutive | Dec/2005-Feb/2008 | Prospective | Children and Adults | 13 | Egypt | 27.25 | 31.13 | 44 | Real time RT-PCR | Nasal, throat, tracheal, bronchoalveolar | 61 |
| Emukele, 2014 | 5898 | 575 | 9.7% | Cross sectional | Rural | Consecutive | Aug/2009–Jul/2012 | Prospective | Children and Adults | 76 | Kenya | 0.062629 | 34.287808 | 1318 | Real time RT-PCR | Nasopharyngeal and Oropharyngeal | 50.8 |
| Enan, 2013 | 334 | 27 | 8.1% | Cross sectional | Unclear/Not described | Consecutive | Jan/2010-Mar/2010 and Jan/2011-Mar/2011 | Prospective | Children and Adults | 92 | Sudan | 15.500654 | 32.559899 | 385 | Real time RT-PCR | Throat |  |
| Fall, 2016 | 5338 | 610 | 11.4% | Cross sectional | Unclear/Not described | Consecutive | Jan/2012-Oct/2015 | Prospective | Children and Adults | 77 | Senegal | 14.497401 | -14.452362 | 49 | Real time RT-PCR | Nasopharyngeal and Oropharyngeal | 49.3 |
| Feikin, 2012 | 1216 | 92 | 7.6% | Cross sectional | Rural | Consecutive | May/2007-Feb2010 | Prospective | Children and Adults | 58.9 | Kenya | -0.179441 | 34.383765 | 1160 | Real time RT-PCR | Nasopharyngeal and Oropharyngeal |  |
| Feikin, 2012 (2) | 520 | 17 | 3.3% | Cross sectional | Rural | Consecutive | Jan/2009-Feb/2010 | Prospective | Children and Adults |  | Kenya | -0.023559 | 37.906193 | 822 | Real time RT-PCR | Nasopharyngeal and Oropharyngeal |  |
| Feikin, 2013 (1) | 408 | 90 | 22.1% | Cross sectional | Rural | Consecutive | Mar/2007–Feb/2010 | Prospective | Children | 100 | Kenya | -0.179441 | 34.383765 | 1160 | Real time RT-PCR | Nasopharyngeal and Oropharyngeal |  |
| Feikin, 2013 (2) | 199 | 50 | 25.1% | Case control | Rural | Consecutive | Jan/2009–Feb/2010 | Prospective | Children | 100 | Kenya | -0.140813 | 34.356017 | 1249 | Real time RT-PCR | Nasopharyngeal and Oropharyngeal |  |
| Fuller, 2013 | 3814 | 479 | 12.6% | Cross sectional | Rural | Consecutive | Aug/2008-Dec/2010 | Prospective | Children and Adults | 54.3 | Kenya | -0.100337 | 34.275499 | 1258 | Real time RT-PCR | Nasopharyngeal and Oropharyngeal |  |
| Ghani, 2012 | 709 | 54 | 7.6% | Cross sectional | Unclear/Not described | Consecutive | Apr/2009-Dec/2009 | Prospective | Children | 100 | South Africa | 33.924868 | 18.424055 | 12 | Real time RT-PCR | Nasopharyngeal/Tracheal/Broncho-alveolar lavage |  |
| Hammitt, 2012 | 805 | 136 | 16.9% | Case control | Rural | Consecutive | Jan/2010-Feb/2010 | Prospective | Children | 100 | Kenya | -3.510651 | 39.90932 | 5 | Real time RT-PCR | Nasopharyngeal and Oropharyngeal | 54.7 |
| Hoffman, 2012 | 937 | 35 | 3.7% | Cross sectional | Rural | Consecutive | Feb/2010-feb/2011 | Prospective | Children | 100 | Madagascar | 21.740959 | 48.042 | 9 | Real time RT-PCR | Nasopharyngeal |  |
| Horton, 2017 | 18247 | 1973 | 10.8% | Cross sectional | Unclear/Not described | Consecutive | 2007-2014 | Prospective | Children and Adults | na | Egypt | 30.04442 | 31.235712 | 26 | Real time RT-PCR | Nasopharyngeal and Oropharyngeal |  |
| Jroundi, 2014 | 684 | 125 | 18.3% | Cross sectional | Unclear/Not described | Consecutive | Nov/2010-Dec/2011 | Prospective | Children | 100 | Morocco | 13.122552 | 5.505313 | 265 | Real time RT-PCR | Nasopharyngeal | 64.3 |
| Jroundi, 2016 | 683 | 124 | 18.2% | Cross sectional | Urban | Consecutive | Nov/2010-Dec/2011 | Prospective | Children | 100 | Morocco | 33.9715904 | -6.8498129 | 78 | Real time RT-PCR | Nasopharyngeal |  |
| Kadjo, 2012 | 470 | 113 | 24.0% | Cross sectional | Urban and Rural | Random | Jan/2009-Dec/2010 | Prospective | Children | 100 | Côte d'Ivoire | 7.539989 | -5.54708 | 240 | Real time RT-PCR | Nasopharyngeal |  |
| Kelly, 2015 | 310 | 89 | 28.7% | Cross sectional | Urban and Rural | Consecutive | Apr/2012-Aug/2014 | Prospective | Children | 100 | Botswana | -24.628208 | 25.923147 | 1000 | Real time RT-PCR | Nasopharyngeal | 55 |
| Kenmoe, 2016 | 347 | 46 | 13.3% | Cross sectional | Urban | Systematic | Sep/2011-Sep/2013 | Prospective | Children | 100 | Cameroon | 3.848033 | 11.502075 | 710 | Real time RT-PCR | Nasopharyngeal | 51.9 |
| Kim, 2011 | 2331 | 328 | 14.1% | Cross sectional | Unclear/Not described | Consecutive | Jun/2009-Aug/2010 | Prospective | Children and Adults | na | Kenya | -0.023559 | 37.906193 | 822 | Real time RT-PCR | Nasopharyngeal and Oropharyngeal | 53.9 |
| Kwofie, 2012 | 128 | 18 | 14.1% | Cross sectional | Urban and Rural | Consecutive | Jan/2008-Dec/2008 | Prospective | Children | 100 | Ghana | -0.277213 | 36.07027 | 1865 | Real time RT-PCR | Nasopharyngeal | 63.3 |
| Lagare, 2015 | 160 | 56 | 35.0% | Cross sectional | Urban and Rural | Consecutive | Jan/2010-Dec/2012 | Prospective | Children | 100 | Niger | 13.506287 | 2.799954 | 252 | Real time RT-PCR | Nasopharyngeal | 56 |
| Lekana-Douki, 2014 | 1041 | 124 | 11.9% | Cross sectional | Urban and Rural | Consecutive | Mar/2010-Jun/2011 | Prospective | Children and Adults | 88.5 | Gabon | -0.3625734 | 12.0213438 | 285.5 | Real time RT-PCR | Nasal | 49.5 |
| Lonngren, 2014 | 569 | 119 | 20.9% | Cross sectional | Unclear/Not described | Consecutive | Jul/2009-Jul/2011 | Prospective | Children | 100 | South Africa | 33.924868 | 18.424055 | 12 | Real time RT-PCR | Nasopharyngeal and Broncho-alveolar lavage | 53.3 |
| Mazur, 2016 | 10128 | 2404 | 23.7% | Cross sectional | Urban and Rural | Consecutive | Feb/2009-Dec/2013 | Prospective | Children | 100 | South Africa | -25.565336 | 30.52791 | 1401 | Real time RT-PCR | Nasopharyngeal |  |
| Meligy, 2016 | 51 | 9 | 17.6% | Cross sectional | Unclear/Not described | Consecutive | Oct/2013-Mar/2014 | Prospective | Children | 100 | Egypt | 30.04442 | 31.235712 | 26 | Real time RT-PCR | Nasopharyngeal | 54.5 |
| Mohamed, 2015 | 168 | 16 | 9.5% | Cross sectional | Unclear/Not described | Consecutive | Jan/2010-Dec/2012 | Prospective | Children and Adults | 73.3 | Kenya | -0.4532293 | 39.6460988 | 146 | Real time RT-PCR | Nasopharyngeal and Oropharyngeal |  |
| Moyes, 2013 | 4293 | 1157 | 27.0% | Cross sectional | Urban and Rural | Consecutive | Jan/2010-Dec/2011 | Prospective | Children | 100 | South Africa | -26.244583 | 30.379412 | 857 | Real time RT-PCR | Nasopharyngeal | 57 |
| Moyes, 2017 | 7796 | 329 | 4.2% | Cross sectional | Urban and Rural | Consecutive | Feb/2009-Dec/2013 | Prospective | Adults | 0 | South Africa | -26.244583 | 30.379412 | 857 | Real time RT-PCR | Nasopharyngeal and Oropharyngeal |  |
| Nakouné, 2013 | 329 | 10 | 3.0% | Cross sectional | Urban and Rural | Consecutive | Jan/2010-Dec/2010 | Prospective | Children | 100 | Central African Republic | 4.8319025 | 18.27050185 | 440 | Real time RT-PCR | Nasopharyngeal |  |
| Ndegwa, 2014 | 140 | 17 | 12.1% | Cross sectional | Urban and Rural | Consecutive | Apr/2010-Sep/2012 | Prospective | Children and Adults | 57.3 | Kenya | -0.69188375 | 35.7949515 | 1425 | Real time RT-PCR | Nasopharyngeal and Oropharyngeal | 60.7 |
| Niang, 2010 | 67 | 13 | 19.4% | Cross sectional | Rural | Consecutive | Jul/2007-Dec/2007 | Prospective | Children | 100 | Senegal | 14.12492 | -16.468563 | 6 | Conventional RT-PCR | Nasopharyngeal | 53.7 |
| Niang, 2017 | 6381 | 266 | 4.2% | Cross sectional | Urban and Rural | Consecutive | Jan/2010-Dec/2015 | Prospective | Children and Adults | 72.2 | Senegal | 14.497401 | -14.452362 | 49 | Real time RT-PCR | Nasopharyngeal and Oropharyngeal | 49.6 |
| Njouom, 2012 | 561 | 32 | 5.7% | Cross sectional | Urban and Rural | Consecutive | Jan/2009-Dec/2009 | Prospective | Children and Adults | 74.2 | Cameroon | 5.4240758 | 10.6650109 | 928.5 | Real time RT-PCR | Throat and Nasopharyngeal |  |
| Nyawanda, 2016 | 3810 | 470 | 12.3% | Cross sectional | Rural | Consecutive | Sep/2009-Aug/2013 | Prospective | Children | 100 | Kenya | 0.0626293 | 34.2878084 | 1318 | Real time RT-PCR | Nasopharyngeal and Oropharyngeal |  |
| Obodai, 2014 | 53 | 32 | 60.4% | Cross sectional | Unclear/Not described | Random | Feb/2006-Nov/2006 | Prospective | Children | 100 | Ghana | 5.603717 | -0.186964 | 60 | Conventional RT-PCR | Nasopharyngeal | 69.8 |
| O'Callaghan-Gordo, 2011 | 475 | 50 | 10.5% | Cross sectional | Rural | Consecutive | Sep/2006-Sep/2007 | Prospective | Children | 100 | Mozambique | -25.891968 | 32.605135 | 52 | Conventional RT-PCR | Nasopharyngeal | 64 |
| Othman, 2016 | 122 | 59 | 48.4% | Cross sectional | Unclear/Not described | Consecutive | Dec/2013-May/2014 | Prospective | Children | 100 | Egypt | 30.04442 | 31.235712 | 26 | Real time RT-PCR | Nasopharyngeal |  |
| Otieno, 2017 | 3561 | 881 | 24.7% | Cross sectional | Unclear/Not described | Consecutive | Sep/2010-Aug/2015 | Prospective | Children | 100 | Kenya | 3.510651 | 39.90932 | 5 | Real time RT-PCR | Nasopharyngeal and Oropharyngeal |  |
| Ouédraogo Yugbaré, 2016 | 209 | 24 | 11.5% | Cross sectional | Urban | Consecutive | Jul/2010-Jun/2011 | Prospective | Children | 100 | Burkina Faso | 12.371428 | -1.51966 | 297 | Real time RT-PCR | Nasopharyngeal |  |
| Ouedraogo, 2014 | 209 | 24 | 11.5% | Cross sectional | Urban | Consecutive | Jul/2010-Jun/2011 | Prospective | Children | 100 | Burkina Faso | 12.371428 | -1.51966 | 297 | Real time RT-PCR | Nasopharyngeal | 58.4 |
| Peterson, 2016 | 1835 | 220 | 12.0% | Cross sectional | Urban | Consecutive | Jan/2011-Dec/2014 | Prospective | Children | 100 | Malawi | -15.677854 | 34.950663 | 719 | Real time RT-PCR | Nasopharyngeal |  |
| Pretorious, 2012 | 8173 | 1169 | 14.3% | Cross sectional | Urban and Rural | Consecutive | Feb/2009-Mar/2012 | Prospective | Children and Adults | na | South Africa | -26.573865 | 29.118588 | 1096 | Real time RT-PCR | Nasopharyngeal and Oropharyngeal | 51.1 |
| Pretorious, 2013 | 13432 | 2182 | 16.2% | Cross sectional | Urban and Rural | Consecutive | Feb/2009-May/2012 | Prospective | Children and Adults | na | South Africa | -26.573865 | 29.118588 | 1096 | Real time RT-PCR | Nasopharyngeal and Oropharyngeal | 51.1 |
| Pretorious, 2016 | 5743 | 616 | 10.7% | Cross sectional | Urban and Rural | Systematic | May/2012-Apr/2015 | Prospective | Children and Adults | 73 | South Africa | -28.26873 | 28.456142 | 1025 | Real time RT-PCR | Nasopharyngeal and Oropharyngeal |  |
| Razanajatovo, 2011 | 313 | 66 | 21.1% | Cross sectional | Urban | Consecutive | Jul/2008-Jun/2009 | Retrospective | Children and Adults |  | Madagascar | -18.8791902 | 47.5079055 | 1249 | Real time RT-PCR | Nasopharyngeal and Oropharyngeal | 44.1 |
| Rowlinson, 2013 | 4683 | 518 | 11.1% | Cross sectional | Urban and Rural | Consecutive | Jun/2009-Jun/2012 | Prospective | Children and Adults | 90.1 | Egypt | 31.042457 | 30.47275 | 10 | Real time RT-PCR | Nasopharyngeal and Oropharyngeal |  |
| Rowlinson, 2016 | 5768 | 669 | 11.6% | Cross sectional | Urban and Rural | Consecutive | Jun/2009-Dec/2013 | Prospective | Children and Adults | 100 | Egypt | 31.042457 | 30.47275 | 10 | Real time RT-PCR | Nasopharyngeal and Oropharyngeal |  |
| Shafik, 2012 | 450 | 107 | 23.8% | Cross sectional | Urban | Consecutive | Nov/2006-Dec/2007 | Prospective | Children | 100 | Egypt | 30.04442 | 31.235712 | 26 | Real time RT-PCR | Nasopharyngeal | 57.4 |
| Simusika, 2015 | 297 | 45 | 15.2% | Cross sectional | Urban | Consecutive | Jan/2011-Dec/2012 | Prospective | Children | 100 | Zambia | -15.3875259 | 28.3228165 | 1253 | Real time RT-PCR | Nasopharyngeal |  |
| Venter, 2011 | 610 | 57 | 9.3% | Cross sectional | Urban and Rural | Consecutive | Jan/2006-Dec/2007 | Retrospective | Children | 100 | South Africa | -25.747868 | 28.229271 | 1370 | Real time RT-PCR | Nasopharyngeal | 52.8 |
| Zar, 2016 | 284 | 66 | 23.2% | Case control | Urban and Rural | Systematic | May/2012-Dec/2014 | Prospective | Children | 100 | South Africa | -33.73423 | 18.962109 | 125 | Real time RT-PCR | Nasopharyngeal | 53 |

ILI: Influenza like infection, RT PCR: reverse transcriptase polymerase chain reaction, SARI: severe acute respiratory infection; na: not available.
